# Physical Activity and Sedentary Behaviors Modify the Association between Melanocortin 4 Receptor Gene Variant and Obesity in Chinese Children and Adolescents

**DOI:** 10.1371/journal.pone.0170062

**Published:** 2017-01-12

**Authors:** Jie-Yun Song, Qi-Ying Song, Shuo Wang, Jun Ma, Hai-Jun Wang

**Affiliations:** Institute of Child and Adolescent Health, School of Public Health, Peking University, Beijing, China; Taipei City Hospital, TAIWAN

## Abstract

Effects of *MC4R* variants in previous Chinese population studies were inconsistent. Gene-environment interactions might influence the effect of *MC4R* variants on obesity, which was still unclear. We performed the study to clarify the association of variants near *MC4R* gene with obesity-related phenotypes and gene-environment interactions in Chinese children and adolescents. Two common variants (rs12970134 and rs17782313) near *MC4R* were genotyped in 2179 children and adolescents aged 7–18 years in Beijing of China. Associations between the variants and obesity-related phenotypes together with gene-environment interactions were analyzed. The A-alleles of rs12970134 were nominally associated with risk of overweight/obesity (Odds Ratios (OR) = 1.21, 95%CI: 1.03–1.44, *P* = 0.025) and BMI (β = 0.33 kg/m^2^, 95%CI: 0.02–0.63, *P* = 0.025), respectively. The rs12970134 was also associated with HDL-C (β = -0.03mmol/L per A-allele, 95%CI: -0.05, -0.01, *P* = 0.013) independent of BMI. In the further analysis, we found the significant interaction of rs12970134 and physical activity/sedentary behaviors on BMI (*P*_*interaction*_ = 0.043). The rs12970134 was found to be associated with BMI only in children with physical activity<1h/d and sedentary behaviors ≥2h/d (BMI: β = 1.27 kg/m^2^, 95%CI: 0.10–2.45, *P* = 0.034). The association was not detected in their counterparts with physical activity≥1h/d or sedentary behaviors <2h/d. We identified the effect of *MC4R* rs12970134 on overweight/obesity and BMI, and we also found physical activity and sedentary behaviors modified the association between the rs12970134 and BMI in Chinese children and adolescents.

## Introduction

The melanocortin-4 receptor (MC4R) is primarily expressed in the central nervous system and a part of melanocortinergic pathway that regulates food intake and controls energy homeostasis [1]. The *MC4R* homozygous knock-out mice had maturity onset obesity, hyperphagia, increased linear growth, hyperinsulinemia, and hyperglycemia, reduced metabolic rate [2]. The role of the central melanocortin system on adiposity by controlling nutrient partitioning and lipid metabolism independent of nutrient intake was reported [3].

Recently, genome-wide association studies (GWAS) demonstrated *MC4R* to be one of the genes contributing to the etiology of common obesity. Loos and colleagues carried out a meta-analysis of GWAS data including 16,876 adults of European descent [4]. A common single nucleotide polymorphism (SNP) rs17782313 at 188 kb downstream of *MC4R* was reported to be strongly associated with body mass index (BMI). The association was replicated in the additional samples of total 60,352 adults and 5,988 children. The effect of rs17782313 on BMI in children was about twice of that observed in adults, indicating rs17782313 was relevant to early-onset obesity. Another GWAS in adults of Indian Asian and European descent found that a common SNP rs12970134 at 154 kb downstream of *MC4R* was associated with waist circumference and insulin resistance [5]. The associations of rs17782313 or rs12970134 with obesity-related phenotypes were confirmed by several subsequent studies in different ethnicity and age groups [6–14]. However, it is not consistent in Chinese population. Despite some studies showed evidence of significant association (Shi et al [7] and Huang et al [15] in adults; Hong et al [16] in young, Wu et al [8] in children), studies by Cheung et al [17] and Tao et al [18] in adults, Wang et al [19] in children revealed non-significant association.

Twin, adoption and family studies have estimated the heritability of BMI ranges from 0.4 to 0.7. But only 1–2% of the BMI variance is explained by the identified variants. One consideration is that if gene-environment interactions are more or less specific for specific populations, the respective genes would potentially escape detection in association studies [20]. So far, several studies found genetic effects varied by diet or physical activity [21–24], suggesting the possible existence of interaction between genes and environmental factors. For *MC4R*, several previous studies analyzed the possible interaction. It showed no interaction between *MC4R* rs17782313 and physical activity on obesity-related traits in Finnish adolescents and French adults [13], Danish adults [9], British adults [24] and Dutch adolescents [25]. However, Xi and colleague [26] found that the association between the *MC4R* rs17782313 and obesity was influenced by sedentary behaviors and physical activity in Chinese children. The interactive results are still differing.

Since the prevalence of childhood obesity has been increasing in the rapidly economically developing Chinese community, China offers an unprecedented opportunity for studying the gene-environmental interaction on childhood obesity. For the inconsistent results from previous studies on interaction between *MC4R* variants and environmental factors, we genotyped two common variants (rs17782313 and rs12970134), analyzed their association with obesity-related phenotypes, and investigated interaction between the SNPs and environmental factors.

## Materials and Methods

### Subjects

The 2179 subjects came from three independent study groups recruited from the urban regions of Beijing, China. The first study group, including 386 obese and 551 nonobese children, came from the study on Adolescent Lipids, Insulin Resistance and Candidate Genes (ALIR) in nine middle schools of Dongcheng District of Beijing. The second study group, including 319 obese and 774 nonobese children, was from the Comprehensive Prevention Project for Overweight and Obese Adolescents (CPOOA) with physical exercise and healthy nutrition as instruments in five elementary and middle schools of Haidian District of Beijing. We used the baseline information in the present study. The third study group was recruited from four schools in Changping District of Beijing (elementary school students aged 7 to 11, middle school students aged 11 to 15). Form the third study group, 149 overweight or obese individuals were selected for DNA samples were available. The ascertainment strategies for the three study groups have been described in detail previously [27–29]. The underweight children were excluded according to the standard for malnutrition of school-age children and adolescents released by National Health and Family Planning Commission of China [30]. We used the uniform BMI percentile criteria for normal-weight, overweight and obese children which was determined in a representative Chinese population [31]. According to the criteria, the children with an age- and sex-specific BMI ≥95^th^ percentile are defined as obese, the ones with a BMI between 85^th^ to 95^th^ percentile are defined as overweight whereas those with a BMI <85^th^ percentile are normal-weight. The individuals with any cardiovascular or metabolic disease were excluded. The three studies were approved by the Ethic Committee of Peking University Health Science Center. Written informed consent was provided by all participants and, in the case of minors, their parents.

### Measure

Anthropometric measurements, including height, weight, waist and hip circumferences, were determined according to standard protocols. Mean systolic and diastolic blood pressures were calculated by averaging three measurements. Fasting venous blood samples were taken for measurement of total cholesterol (TC), triglyceride (TG), low-density lipoprotein cholesterol (LDL-C), high-density lipoprotein cholesterol (HDL-C) and fasting glucose using a biochemical auto-analyzer (Hitachi 7060, Tokyo, Japan). Fasting insulin was determined by the radio-immunoassay method (Beijing North Institute of Biological Technology, Beijing, China). The sex- and age-specific BMI standard deviation score (BMI-SDS) was calculated by using the growth reference data of the World Health Organization for children and adolescents aged 5–19 years [32].

### Questionnaire

A validated questionnaire was used to investigate environmental factors of childhood obesity, including dietary behaviors and physical activity in the CPOOA study, including 1093 individuals [33, 34]. The questionnaires were completed by children and their parents or guardians. The frequencies of dietary behavior and physical activity level during the last week before investigation were reported. In the questionnaire we ask how often the participants consumed cereal/wheat products, meat/fish/soybeans/egg, fruits and vegetables, milk or yogurt, high calorie foods (fried chips/cakes/cookies), soft drinks. They could choose an option from “Never, 1–3 times, 4–6 times, daily, twice per day, 3 times per day, or more than 3 times per day”. The level of physical activity was evaluated by the daily time spent on physical activity and the time spent on sedentary behaviors per day (including watching television/video, playing computer). The option were “Never, 0–0.5h per day, 0.5-1h per day, 1-2h per day, 2-3h per day, 3-4h per day, more than 4h per day”. Each variable was classified into two categories based on national recommendation of nutrition and physical activity for Chinese children [35].

### Genotyping

Genotyping of two common variants (rs12970134 and rs17782313) was carried out with tetra-primer amplification refractory mutation system analysis (tetra-primer ARMS-PCR) [36]. The primers and enzymes for genotyping the above variants can be obtained from the authors. The rs12970134 was genotyped in all 2179 samples and rs17782313 was genotyped in the first two study groups with sample size of 2030. The call rates of the two variants were 99.5% and 100% separately.

PCR products of different genotypes were clearly distinguished on 2.5% agarose gels stained with ethidium bromide. For reference PCR of individuals with genotypes identified by sequencing were included in every run. At least two experienced individuals independently assigned the genotypes. Discrepancies were solved unambiguously either by reaching consensus or by repeating. We genotyped 5% of samples twice for quality control and the genotyping concordance rate was 100%.

### Statistical analyses

The genotype data of the normal-weight group was tested for deviation from Hardy—Weinberg equilibrium with χ^2^ test. The relationships between SNPs and quantitative variables were tested by using linear regression adjusted for age, sex and study groups. Logisitic regression adjusted for the same covariates was used to calculate the odds ratios (ORs) of a risk allele for obesity. All analyses were performed under an additive genetic model. Stratified and joint association analyses were conducted to evaluate the combined effects of gene and environmental factors. The above statistical analyses were performed with SPSS 18.0 software (SPSS Inc., Chicago, IL, USA).

Estimation of linkage disequilibrium (LD) between variants was tested by calculating r^2^ with Haploview 4.1.

## Results

### Association between common variants near *MC4R* and obesity-related phenotypes

The detailed characteristics of each study group were shown in Table 1. In the first two independent studies, there were more boys in overweight and obese groups; the average age of overweight and obese individuals were younger; and the different of weight, waist circumstance and BMI were significant among different groups (*P*<0.001). The difference of physical activity level and sedentary behaviors were not statistically significant (*P*>0.05).

**Table 1:** General characteristics of the study groups.

	Normal-weight group (n = 607)	Overweight group (n = 730)	Obese group (n = 842)	P-value
	**Study 1**			
n	151	400	386	
Male (%)	88(58.3)	250(62.5)	269(69.7)	
Female (%)	63(41.7)	150(37.5)	117(30.3)	0.020
Age (year)	14.81±0.75	14.63±0.56	14.6±0.55	<0.001
Height (cm)	166.8±8.02	167.61±7.68	170.41±7.61	<0.001
Weight (kg)	56.87±7.20	70.62±7.13	87.47±12.82	<0.001
Waist circumstance (cm)	69.4±6.55	80.87±4.78	93.05±9.15	<0.001
BMI (kg/m2)	20.41±1.83	25.08±1.00	30.02±3.07	<0.001
	**Study2**			
n	456	318	319	
Male (%)	196(43.0)	200(62.9)	215(67.4)	
Female (%)	260(57.0)	118(37.1)	104(32.6)	<0.001
Age(year)	11.8±3.15	11.54±2.63	10.73±2.51	<0.001
Height (cm)	152.69±16.25	154.84±14.67	153.45±13.93	0.150
Weight (kg)	43.86±13.57	54.88±14.72	62.37±18.35	<0.001
Waist circumstance (cm)	63.15±7.45	73.98±7.9	82.2±10.73	<0.001
BMI (kg/m2)	18.24±2.46	22.33±2.34	25.82±3.64	<0.001
Daily physical activity time				0.596
≥1h/d	220(53.0)	150(56.8)	144(55.6)	
<1h/d	195(47.0)	114(43.2)	115(44.4)	
Sedentary behaviors ^a^				0.890
<2h/d	268(64.0)	167(62.8)	163(62.2)	
≥2h/d	151(36.0)	99(37.2)	99(37.8)	
Physical activity level & Sedentary behaviors				0.723
Physical activity≥1h/d or sedentary behaviors < 2h/d	72(17.2)	50(18.9)	43(16.3)	
Physical activity <1h/d and sedentary behaviors ≥2h/d	347(82.8)	214(81.1)	220(83.7)	
	**Study 3**			
n	-	12	137	
Male (%)	-	9(75.0)	96(70.1)	
Female (%)	-	3(25.0)	41(29.9)	-
Age(year)	-	9.75±1.48	9.34±1.66	-
Height (cm)	-	146.54±10.61	147.53±10.62	-
Weight (kg)	-	46.25±9.01	56.94±14.44	-
Waist circumstance (cm)	-	73.39±5.55	83.03±9.88	-
BMI (kg/m2)	-	21.31±1.50	25.74±3.52	-

BMI: body mass index; SDS: standard deviation score.

^a^ Sedentary behaviors included watching television, playing computer and video game.

Without normal-weight individuals included in the third study, the different between groups were not tested. No deviation from Hardy-Weinberg equilibrium was observed for rs12970134 and rs1772313 in normal-weight children (*P* = 0.171 and 0.110, respectively). The minor allele frequencies of rs12970134 and rs17782313 in normal-weight children were 18.89% and 21.25%, respectively. The two variants were found to be in weak linkage disequilibrium (LD) (r^2^ = 0.63) in the study groups.

Analyzing the association between the rs12970134 and BMI, we found a nominally significant association. The carriers of A-allele had higher BMI than homozygous subjects of G-allele, with an increase of 0.33 kg/m^2^ (95%CI: 0.02–0.63, *P* = 0.034) for each additional A-allele (Table 2). The rs12970134 A-allele was also associated with a higher prevalence of overweight/obesity (OR = 1.20, 95%CI: 1.02–1.42, *P* = 0.025). For rs17782313, we did not find significant association with neither BMI nor risk of overweight/obesity. We further analyzed the relationships between the two variants and obesity-related phenotypes with linear regression analysis adjusted for age, sex and study groups under an additive genetic model (Table 3). The A-allele of rs12970134 was associated with higher hip circumstance (β = 0.66cm, 95%CI: 0.04–1.28, *P* = 0.038), lower HDL-C (b = -0.03mmol/L, 95%CI: -0.06, -0.01, *P* = 0.002) and higher body fat percentage (β = 0.70%, 95%CI: 0.002–1.40, *P* = 0.049). After additionally adjusted for BMI, the association with HDL-C was still significant (β = -0.03mmol/L, 95%CI: -0.05, -0.01, *P* = 0.013). No significant association was found between the two variants and other obesity-related phenotypes including blood pressure, fasting glucose, insulin, etc.

**Table 2:** Association between rs12970134 and rs17782313 and anthropometric measures.

Variant	Phenotypes	*MC4R* genotypes			Adjusted	
					β or OR (95%CI)	*P*-value
**rs12970134**		**GG (n = 1349)**	**GA (n = 718)**	**AA(n = 102)**		
	**BMI, kg/m**^**2**^ **(Mean (SD))**	23.75(4.78)	24.23(4.75)	23.94(4.24)	0.33(0.02, 0.63)	0.034
	**Overweight/ obesity (%)**	1022(70.1)	576(75.5)	79(73.5)	1.21(1.03, 1.44)	0.025
**rs17782313**		**TT(n = 1202)**	**TC(n = 702)**	**CC(n = 126)**		
	**BMI, kg/m**^**2**^ **(Mean(SD))**	23.70(4.90)	23.98(4.70)	24.05(4.29)	0.06(-0.22, 0.34)	0.687
	**Overweight /obesity (%)**	819(68.1)	512(72.9)	92(73.0)	1.14(0.97, 1.35)	0.120

BMI, body mass index; OR, odds ratio; CI, confidence interval.

Effect sizes and *P*-values were estimated under additive genetic model adjusted for age, sex and study group in the combined analyses.

**Table 3:** The association study of rs12970134 and rs17782313 with obesity-related phenotypes.

	rs12970134				rs17782313			
	n	Estimate (95%CI) ^a^	*P*-value ^a^	*P*-value ^b^	n	Estimate (95%CI) ^a^	*P*-value ^a^	*P*-value ^b^
**Height(cm)**	2169	0.28(-0.22, 0.79)	0.274	-	2030	0.54(0.04, 1.03)	0.035	-
**Weight(kg)**	2169	0.88(-0.09, 1.85)	0.074	-	2030	0.40(-0.53, 1.32)	0.399	-
**BMI-SDS**	2169	0.07(-0.13, 0.14)	0.100	-	2030	0.03(-0.40, 0.11)	0.361	-
**Waist circumference(cm)**	2156	0.73(-0.06, 1.52)	0.070	0.744	2017	0.24(-0.50, 0.98)	0.521	0.476
**Hip circumference(cm)**	2154	0.66(0.04, 1.28)	**0.038**	0.783	2015	0.31(-0.28, 0.90)	0.299	0.134
**Waist-to-Hip Ratio**	2154	0.002(-0.002, 0.01)	0.333	0.634	2015	0.002(-0.002, 0.002)	0.911	0.856
**Total Cholestestrol(mmol/L)**	2169	-0.03(-0.09, 0.02)	0.242	0.126	2030	-0.03 (-0.097, 0.02)	0.190	0.172
**Triglyceride(mmol/L)**	2169	0.03(-0.01,0.07)	0.152	0.456	2030	-0.01(-0.05, 0.02)	0.472	0.363
**LDL-C(mmol/L)**	2169	0.01(-0.04, 0.06)	0.728	0.683	2030	0.002(-0.047, 0.051)	0.943	0.962
**HDL-C(mmol/L)**	2169	-0.03(-0.06, -0.01)	**0.002**	**0.013**	2030	-0.02 (-0.03, 0.003)	0.107	0.119
**Systolic BP(mmHg)**	2159	0.6(-0.40, 1.56)	0.237	0.941	2021	0.30(-0.64, 1.24)	0.533	0.602
**Diastolic BP(mmHg)**	2159	1.07(-0.19, 2.34)	0.096	0.329	2021	0.62(-0.43, 1.67)	0.245	0.265
**Fasting glucose(mmol/L)**	2169	-0.01(-0.05, 0.03)	0.653	0.861	2030	-0.01(-0.04, 0.03)	0.741	0.719
**Body fat percentage (%)**	1061	0.70(0.002, 1.40)	**0.049**	0.428	1071	0.36(-0.32, 1.05)	0.298	0.896
**Metabolic syndrome**	2153	1.05(0.90–1.23)	0.523	0.596	2015	0.91(0.77–1.06)	0.210	0.168

BMI, body mass index; BMI-SDS, BMI standard deviation score; LDL-C, low-density lipoprotein cholesterol; HDL-C, high-density lipoprotein cholesterol; BP, blood pressure; CI, confidence interval.

^a^ Effect sizes and *P*-values were calculated adjusted for age, sex and study groups.

^b^ *P*-values were calculated additionally adjusted for BMI.

### Gene-lifestyle interaction

We investigated the combined effect of physical activity, sedentary behaviors and *MC4R* variants on BMI and risk of overweight/obesity. In stratified subgroups of different level of physical activity or sedentary behaviors, we only found the risk A-allele of rs12970134 variant was marginally associated with increased BMI in children who participated in sedentary behaviors≥2h/day (β = 0.74kg/m^2^, 95%CI: 0.01–1.47, *P* = 0.048, Table 4). In the further analysis, we found a statistically significant interaction between physical activity/sedentary behaviors and rs12970134 on BMI (*P*-interaction = 0.043). The rs12970134 variant was only significantly associated with BMI in children with physical activity <1h/d and sedentary behaviors ≥2h/d (β = 1.27kg/m^2^, 95%CI: 0.10–2.45, *P* = 0.034). While in children with physical activity ≥2h/d or sedentary behaviors <2h/d, the rs12970134 was not associated with BMI (*P* = 0.628).

**Table 4:** Association of rs12970134 with BMI stratified by physical activity and sedentary behaviors.

	Mean BMI				
	GG(n = 603)	GA(n = 279)	AA(n = 48)	β (95%CI)	*P*-value
Physical activity					
≥1h/d	22.06±4.34	21.77±4.73	22.77±2.68	0.16(-0.54,0.87)	0.646
<1h/d	20.934.28	21.414.18	21.854.28	0.34(-0.18,0.87)	0.196
Sedentary behaviors					
<2h/d	21.13±4.26	20.80±4.16	21.75±4.01	-0.02(-0.53,0.5)	0.948
≥2h/d	22.05±4.42	22.88±4.52	22.85±3.47	0.74(0.01,1.47)	**0.048**
Physical activity/Sedentary behaviors					
Physical activity ≥ 1h/d or sedentary behaviors < 2h/d	21.27±4.35	21.15±4.25	21.92±3.96	0.11(-0.34,0.56)	0.628
Physical activity < 1h/d and sedentary behaviors ≥2h/d	22.30±4.25	23.64±4.62	24.24±1.03	1.27(0.10,2.45)	**0.034**

Effects on BMI were analyzed by using linear regression adjusted for age and sex.

Sedentary behaviors included watching television/video, playing computer.

We tested the combined effect of rs12970134 and diet factors. Individual diet factor did not clearly modify the effect of rs12970134 on BMI, except estimates were lightly increased for individuals with vegetables consumption <2 times/d (see S1 Table).

Likewise, we found a similar trend that lifestyle modified rs12970134 effect on central obesity or body fat percentage. Point estimates were lightly increased for individuals with vegetables consumption <2 times/d, high calorie foods consumption last week, and sedentary behaviors ≥2h/d. Also the effect was slightly bigger in physical activity <1h/d combined with sedentary behaviors ≥2h/d group than sedentary behaviors ≥2h/d group. For metabolic syndrome, we did not find the similar effect (see S1 Fig).

To further test whether the observed interaction between rs12970134 and physical activities and sedentary behaviors were influenced by the missing data, we performed the sensitivity analyses by replacing the missing data. The results were very similar (data not shown).

## Discussion

In the present study, we validated the effect of rs12970134 on BMI and risk of overweight/obesity in Chinese children. Additionally, we found the effect could be modified by dietary factors, physical activity and sedentary behaviors.

The rs17782313 and rs12970134 variants, mapped 188kb and 154kb downstream of *MC4R* gene respectively, were found to be associated with BMI and obesity-related phenotypes in previous GWAS studies [4, 5]. The rs12970134 was in high LD with rs17782313 (r^2^ = 0.811) in CEU Hapmap based on samples of Utah residents with ancestry from northern and western Europe. However, the two variants were found to be in weak LD (r^2^ = 0.63) in our Chinese study group. So we analyzed the effects of rs12970134 and rs17782313 respectively.

The minor allele frequencies for both variants were 18.89% and 21.25% in normal-weight children, which were similar to that reported in Chinese population previously (14–21% in normal-weight population). We found the rs12970134 variant was significantly associated with BMI and risk of overweight/obesity. In addition, we found rs12970134 risk allele was marginally associated with other adiposity phenotypes such as higher hip circumstance and higher fat percentage. The associations disappeared after further adjusted for BMI, indicating the effects were most likely explained by the association with fatness. However, the effect on HDL-C was independently of BMI. The rs17782313 variant showed a similar trend, but did not reach statistical significance.

The two variants were investigated in many independent studies with inconsistent results. It is possible due to different ethnicity and age of different populations. A meta-analysis for rs17782313 variant (or proxy), including 80,957 cases and 220,223 controls, indeed showed significant between-study heterogeneity (I^2^ = 54.8%, *P* for heterogeneity<0.001). In the further analysis, heterogeneity remained significant even in the subgroups according to different ethnicity and age (*P* for heterogeneity<0.05) [37]. It suggested that some confounding factors such as lifestyle factors other than ethnicity and age might impact the effect of these variants.

Several previous studies explored whether *MC4R* and lifestyle factors interacted on obesity. In a Danish population based study, interactions between six frequent *MC4R* variants (rs17782313, rs17700633, rs12970134, rs477181, rs502933, rs4450508) and self-reported physical activity on BMI and waist circumference were analyzed, but no significant interaction was observed [9]. A negative interaction was also reported in Finnish adolescents and French adults between rs17782313 and physical activity on BMI z-score [13]. Corella and colleagues did not find any significant interaction in Spain adults, however, observed a trend that association between rs17782313 risk allele and higher BMI or risk of obesity was greater of magnitude in sedentary subjects than in active subjects [38]. In a Chinese children study, Xi and colleagues found rs17782313 was associated with risk of obesity only in children with sedentary behaviors ≥2 hours/day or participated in moderate or low physical activity (reference group: non-risk allele with high physical activity and sedentary behaviors <2 hours/day, P for trend <0.001) [26]. In line with the previous studies, we identified physical activity alone did not modify the association between rs12970134 variant and BMI or body adiposity, while sedentary behaviors slightly strengthened the rs12970134 effect. We furthered to find physical activity level combined with sedentary behaviors influenced the effect of rs12970134 variant. The rs12970134 A-allele was significantly associated with higher BMI and body adiposity only in children with physical activity <1h/d and sedentary behaviors ≥2h/d. We also found vegetables and high calorie foods consumption marginally influenced effect of rs12970134. Without adjustment for total energy intake, the dietary factors need to be carefully evaluated. The data suggested the unhealthy lifestyle strengthen the contribution of *MC4R* variant to obesity.

Some studies demonstrated physical activity may regulate gene expression in lipogenesis and fatty acid synthesis [39–41]. *MC4R* is expressed primarily in the central nervous system, and included in melanocortinergic pathway that regulates food intake and controls energy homeostasis [39–41]. The *MC4R* homozygous knock-out mice had hyperphagia [2]. It is not clear whether physical activities regulate MC4R expression. Further functional studies are necessary to investigate the mechanism of gene-environment effect.

The main strength of our study was we performed the analysis in a population with multiple phenotypes and lifestyles factorsinformation, based on which the true effects of *MC4R* variants were analyzed. Besides, to examine the robustness of the findings in the present study, we analyzed the combined effect on other indicators of adiposity. We found the similar effect on waist circumstance and body fat percentage, which strengthened the evidence of combined effect of rs12970134 on adiposity.

However, several limitations should be noted. First, environmental factors in our study were measured by self-reported questionnaire, which might be imprecise. In addition, assessment with frequency questionnaire would loss information. For example, it does not assess portion size, information from food intake frequency is limited in accuracy and completeness. Second, confounding by other unmeasured or unknown factors might exist, such as parental factors, family income and so on. Besides gene lifestyle interactions may be confounded by latent variables such as intake of total energy, which was not available in our study. Third, interaction effect estimates may not reflect causal processes with the case-control data. Reverse causality is a further concern. Therefore, further investigation is necessary in large cohort studies with well-collected information and long term follow-up.

## Conclusion

In conclusion, we found rs12970134 variant was nominally associated with BMI, risk of overweight/obesity, hip circumstance and body fat percentage. The rs12970134 was also associated with HDL-C independent of BMI. Physical activity and sedentary behaviors were identified in modifying the effect of rs12970134 variant on BMI and risk of overweight/obesity in Chinese children. The study results help to clarify the effects of *MC4R* genetic variants on childhood obesity.

## Supporting Information

S1 Table Association of rs12970134 with BMI stratified by dietary behaviors. (DOCX)

S1 Fig Association of rs12970134 with waist circumstance, body fat percentage, overweight/obesity and metabolic syndrome stratified by lifestyles. (DOCX)

## References

[1] Tao YX. The melanocortin-4 receptor: physiology, pharmacology, and pathophysiology. Endocr Rev 2010; 31: 506–43. 10.1210/er.2009-0037 20190196 PMC3365848

[2] Huszar D, Lynch CA, Fairchild-Huntress V, Dunmore JH, Fang Q, Berkemeier LR, et al Targeted disruption of the melanocortin-4 receptor results in obesity in mice. Cell 1997; 88: 131–41. 9019399 10.1016/s0092-8674(00)81865-6

[3] Nogueiras R, Wiedmer P, Perez-Tilve D, Veyrat-Durebex C, Keogh JM, Sutton GM, et al The central melanocortin system directly controls peripheral lipid metabolism. J Clin Invest 2007; 117: 3475–88. 10.1172/JCI31743 17885689 PMC1978426

[4] Loos RJ, Lindgren CM, Li S, Wheeler E, Zhao JH, Prokopenko I, et al Common variants near MC4R are associated with fat mass, weight and risk of obesity. Nat Genet 2008; 40: 768–75. 10.1038/ng.140 18454148 PMC2669167

[5] Chambers JC, Elliott P, Zabaneh D, Zhang W, Li Y, Froguel P, et al Common genetic variation near MC4R is associated with waist circumference and insulin resistance. Nat Genet 2008; 40: 716–8. 10.1038/ng.156 18454146

[6] Liu G, Zhu H, Lagou V, Gutin B, Barbeau P, Treiber FA, et al Common variants near melanocortin 4 receptor are associated with general and visceral adiposity in European- and African-American youth. J Pediatr 2010; 156: 598–605. 10.1016/j.jpeds.2009.10.037 20070976 PMC4018229

[7] Shi J, Long J, Gao YT, Lu W, Cai Q, Wen W, et al Evaluation of genetic susceptibility loci for obesity in Chinese women. Am J Epidemiol 2010; 172: 244–54. 10.1093/aje/kwq129 20616199 PMC2917056

[8] Wu L, Xi B, Zhang M, Shen Y, Zhao X, Cheng H, et al Associations of six single nucleotide polymorphisms in obesity-related genes with BMI and risk of obesity in Chinese children. Diabetes 2010; 59: 3085–9. 10.2337/db10-0273 20843981 PMC2992769

[9] Zobel DP, Andreasen CH, Grarup N, Eiberg H, Sorensen TI, Sandbaek A, et al Variants near MC4R are associated with obesity and influence obesity-related quantitative traits in a population of middle-aged people: studies of 14,940 Danes. Diabetes 2009; 58: 757–64. 10.2337/db08-0620 19073769 PMC2646077

[10] Meyre D, Delplanque J, Chevre JC, Lecoeur C, Lobbens S, Gallina S, et al Genome-wide association study for early-onset and morbid adult obesity identifies three new risk loci in European populations. Nat Genet 2009; 41: 157–9. 10.1038/ng.301 19151714

[11] Thorleifsson G, Walters GB, Gudbjartsson DF, Steinthorsdottir V, Sulem P, Helgadottir A, et al Genome-wide association yields new sequence variants at seven loci that associate with measures of obesity. Nat Genet 2009; 41: 18–24. 10.1038/ng.274 19079260

[12] Willer CJ, Speliotes EK, Loos RJ, Li S, Lindgren CM, Heid IM, et al Six new loci associated with body mass index highlight a neuronal influence on body weight regulation. Nat Genet 2009; 41: 25–34. 10.1038/ng.287 19079261 PMC2695662

[13] Cauchi S, Stutzmann F, Cavalcanti-Proenca C, Durand E, Pouta A, Hartikainen AL, et al Combined effects of MC4R and FTO common genetic variants on obesity in European general populations. J Mol Med (Berl) 2009; 87: 537–46. 19255736 10.1007/s00109-009-0451-6

[14] Cha S, Koo I, Park BL, Jeong S, Choi SM, Kim KS, et al Genetic Effects of FTO and MC4R Polymorphisms on Body Mass in Constitutional Types. Evid Based Complement Alternat Med 2011; 2011: 106390 10.1093/ecam/nep162 19822564 PMC3094695

[15] Huang W, Sun Y, Sun J. Combined effects of FTO rs9939609 and MC4R rs17782313 on obesity and BMI in Chinese Han populations. Endocrine 2011; 39: 69–74. 10.1007/s12020-010-9413-6 21063808

[16] Hong J, Shi J, Qi L, Cui B, Gu W, Zhang Y, et al Genetic susceptibility, birth weight and obesity risk in young Chinese. Int J Obes (Lond) 2013; 37: 673–7. 22710927 10.1038/ijo.2012.87

[17] Cheung CY, Tso AW, Cheung BM, Xu A, Ong KL, Fong CH, et al Obesity susceptibility genetic variants identified from recent genome-wide association studies: implications in a chinese population. J Clin Endocrinol Metab 2010; 95: 1395–403. 10.1210/jc.2009-1465 20061430

[18] Tao L, Zhang Z, Chen Z, Zhou D, Li W, Kan M, et al A Common variant near the melanocortin 4 receptor is associated with low-density lipoprotein cholesterol and total cholesterol in the Chinese Han population. Mol Biol Rep 2012; 39: 6487–93. 10.1007/s11033-012-1476-4 22350153

[19] Wang J, Mei H, Chen W, Jiang Y, Sun W, Li F, et al Study of eight GWAS-identified common variants for association with obesity-related indices in Chinese children at puberty. Int J Obes (Lond) 2012; 36: 542–7. 22083549 10.1038/ijo.2011.218

[20] Hebebrand J, Volckmar AL, Knoll N, Hinney A. Chipping away the 'missing heritability': GIANT steps forward in the molecular elucidation of obesity—but still lots to go. Obes Facts 2010; 3: 294–303. 10.1159/000321537 20975295 PMC6452141

[21] Qi Q, Chu AY, Kang JH, Huang J, Rose LM, Jensen MK, et al Fried food consumption, genetic risk, and body mass index: gene-diet interaction analysis in three US cohort studies. BMJ 2014; 348: g1610 10.1136/bmj.g1610 24646652 PMC3959253

[22] Qi Q, Chu AY, Kang JH, Jensen MK, Curhan GC, Pasquale LR, et al Sugar-sweetened beverages and genetic risk of obesity. N Engl J Med 2012; 367: 1387–96. 10.1056/NEJMoa1203039 22998338 PMC3518794

[23] Kilpelainen TO, Qi L, Brage S, Sharp SJ, Sonestedt E, Demerath E, et al Physical activity attenuates the influence of FTO variants on obesity risk: a meta-analysis of 218,166 adults and 19,268 children. PLoS Med 2011; 8: e1001116 10.1371/journal.pmed.1001116 22069379 PMC3206047

[24] Li S, Zhao JH, Luan J, Ekelund U, Luben RN, Khaw KT, et al Physical activity attenuates the genetic predisposition to obesity in 20,000 men and women from EPIC-Norfolk prospective population study. PLoS Med 2010; 7. 10.1371/journal.pmed.1000332 PMC2930873 20824172

[25] Liem ET, Vonk JM, Sauer PJ, van der Steege G, Oosterom E, Stolk RP, et al Influence of common variants near INSIG2, in FTO, and near MC4R genes on overweight and the metabolic profile in adolescence: the TRAILS (TRacking Adolescents' Individual Lives Survey) Study. Am J Clin Nutr 2010; 91: 321–8. 10.3945/ajcn.2009.28186 20007308

[26] Xi B, Wang C, Wu L, Zhang M, Shen Y, Zhao X, et al Influence of physical inactivity on associations between single nucleotide polymorphisms and genetic predisposition to childhood obesity. Am J Epidemiol 2011; 173: 1256–62. 10.1093/aje/kwr008 21527513

[27] Wang HJ, Zhang H, Zhang SW, Pan YP, Ma J. Association of the common genetic variant upstream of INSIG2 gene with obesity related phenotypes in Chinese children and adolescents. Biomed Environ Sci 2008; 21: 528–36. 10.1016/S0895-3988(09)60013-1 19263810

[28] Wang D, Ma J, Zhang S, Hinney A, Hebebrand J, Wang Y, et al Association of the MC4R V103I polymorphism with obesity: a Chinese case-control study and meta-analysis in 55,195 individuals. Obesity (Silver Spring) 2010; 18: 573–9. 19696756 10.1038/oby.2009.268

[29] Li XH, Lin S, Guo H, Huang Y, Wu L, Zhang Z, et al Effectiveness of a school-based physical activity intervention on obesity in school children: a nonrandomized controlled trial. BMC Public Health 2014; 14: 1282 10.1186/1471-2458-14-1282 25510313 PMC4320634

[30] Screening standard for malnutrition of school-age children and adolescents. National Health and Family Planning Commission of China. WS/T 456–2014.

[31] Ji CY. Report on childhood obesity in China (1)—body mass index reference for screening overweight and obesity in Chinese school-age children. Biomed Environ Sci 2005; 18: 390–400. 16544521

[32] WHO: Growth reference data for 5–19 years. http://www.who.int/growthref/tools/en/. Accessed: 2016.

[33] Ma J, Li SS, Wang HJ, Zhang SW, Zhang B. Eating Behaviors of Overweight and Normal -weight Children and Adolescents in Five Cities. Chinese Jounal of School Health 2009; 30: 201–3.

[34] Ma J, Wu SS, Li BH, Wang HJ, Zhang SW, Zhang B. Investigation on Physical Activity Among Children and Adolescents with Different Nutritional Status in Five Cities in China. Chinese Jounal of School Health 2009; 30: 214–7.

[35] Chen CM. Prevention and control guidelines of overweight and obesity for Chinese school children and adolescents. 1st ed. Beijng: People's Medical Publishing House; 2007, p.36–47.

[36] Ye S, Dhillon S, Ke X, Collins AR, Day IN. An efficient procedure for genotyping single nucleotide polymorphisms. Nucleic Acids Res 2001; 29: E88 11522844 10.1093/nar/29.17.e88 PMC55900

[37] Xi B, Chandak GR, Shen Y, Wang Q, Zhou D. Association between common polymorphism near the MC4R gene and obesity risk: a systematic review and meta-analysis. PLoS One 2012; 7: e45731 10.1371/journal.pone.0045731 23049848 PMC3458070

[38] Corella D, Ortega-Azorin C, Sorli JV, Covas MI, Carrasco P, Salas-Salvado J, et al Statistical and biological gene-lifestyle interactions of MC4R and FTO with diet and physical activity on obesity: new effects on alcohol consumption. PLoS One 2012; 7: e52344 10.1371/journal.pone.0052344 23284998 PMC3528751

[39] Walker WA, Blackburn G. Symposium introduction: nutrition and gene regulation. J Nutr 2004; 134: 2434S–2436S. 10.1093/jn/134.9.2434S 15333738

[40] Fiebig R, Gore MT, Ji LL. Exercise attenuates nuclear protein binding to gene regulatory sequences of hepatic fatty acid synthase. J Appl Physiol (1985) 1999; 87: 1009–15. 10484571 10.1152/jappl.1999.87.3.1009

[41] Goodridge AG. Dietary regulation of gene expression: enzymes involved in carbohydrate and lipid metabolism. Annu Rev Nutr 1987; 7: 157–85. 10.1146/annurev.nu.07.070187.001105 3300731

