# Supplementary material for: Physical Activity and Sedentary Behaviors Modify the Association between Melanocortin 4 Receptor Gene Variant and Obesity in Chinese Children and Adolescents
Source: PLoS One. 2017 Jan 12;12(1):e0170062. doi: 10.1371/journal.pone.0170062 (PMC5231371; doi:10.1371/journal.pone.0170062)
Supplement: S1 Table — (DOCX) [file pone.0170062.s001.docx]

S1 Table. Association of rs12970134 with BMI stratified by dietary behaviors

|  | Mean BMI | | |  |  |
| --- | --- | --- | --- | --- | --- |
|  | GG | GA | AA | β (95%CI) | *P*-value |
| Cereal/wheat products |  |  |  |  |  |
| <2/d | 21.42±4.18 | 21.49±4.46 | 22.33±3.71 | 0.21(-0.32,0.74) | 0.433 |
| ≥2/d | 21.55±4.59 | 21.81±4.49 | 22.10±4.35 | 0.51(-0.23,1.24) | 0.177 |
| Meat/fish/soybeans/egg |  |  |  |  |  |
| <2/d | 21.54±4.11 | 21.82±4.93 | 22.44±3.67 | 0.35(-0.30,0.99) | 0.292 |
| ≥2/d | 21.33±4.35 | 21.36±4.15 | 22.29±3.83 | 0.29(-0.27,0.86) | 0.309 |
| Fruits |  |  |  |  |  |
| <2/d | 21.63±4.28 | 21.23±4.45 | 22.14±4.40 | 0.6(-0.13,1.33) | 0.109 |
| ≥2/d | 21.00±4.38 | 22.41±4.42 | 22.67±2.63 | 0.12(-0.41,0.65) | 0.662 |
| Vegetables |  |  |  |  |  |
| <2/d | 22.06±4.31 | 21.10±4.52 | 22.36±3.58 | 0.54(0.03,1.05) | 0.038 |
| ≥2/d | 21.17±4.30 | 21.78±4.43 | 22.41±3.89 | -0.26(-1.1,0.57) | 0.533 |
| Milk or yogurt |  |  |  |  |  |
| 1/d | 21.36±4.38 | 21.46±4.39 | 22.37±3.80 | 0.54(-0.36,1.43) | 0.237 |
| <1/d | 21.67±4.15 | 22.02±4.66 | 22.38±3.51 | 0.26(-0.23,0.75) | 0.295 |
| Fried chips/cakes/cookies |  |  |  |  |  |
| No | 21.95±4.01 | 22.14±4.82 | 22.40±3.66 | -0.15(-1.08,0.78) | 0.751 |
| Yes | 21.28±4.39 | 21.40±4.36 | 22.36±3.77 | 0.43(-0.05,0.92) | 0.078 |
| Soft drink |  |  |  |  |  |
| No | 20.92±4.22 | 21.33±3.88 | 21.83±4.40 | 0.33(-0.29,0.96) | 0.296 |
| Yes | 21.82±4.38 | 21.79±4.77 | 22.39±3.41 | 0.28(-0.3,0.85) | 0.343 |
