# Supplementary material for: Physical Activity and Sedentary Behaviors Modify the Association between Melanocortin 4 Receptor Gene Variant and Obesity in Chinese Children and Adolescents
Source: PLoS One. 2017 Jan 12;12(1):e0170062. doi: 10.1371/journal.pone.0170062 (PMC5231371; doi:10.1371/journal.pone.0170062)
Supplement: S1 Fig — (DOCX) [file pone.0170062.s002.docx]

| Cereal/wheat products | <2/d |
| --- | --- |
|  | ≥2/d |
| Meat/fish/  soybeans/egg | <2/d |
|  | ≥2/d |
| Fruits | ≥2/d |
|  | <2/d |
| Vegetables | ≥2/d |
|  | <2/d |
| Milk or yogurt | 1/d |
|  | <1/d |
| Fried chips/ cakes/cookies | No |
|  | Yes |
| Soft drink | No |
|  | Yes |
| Physical activity | ≥1h/d |
|  | <1h/d |
| Sedentary behaviors | <2h/d |
|  | ≥2h/d |
| Physical activity/  Sedentary behaviors | PA≥1h/d or SB<2h/d |
|  | PA<1h/d and SB≥2h/d |

Waist circumstance (cm) Body fat percentage (%)

S1 Fig. Association of rs12970134 with waist circumstance, body fat percentage, overweight/obesity and metabolic syndrome stratified by lifestyles

PA: Physical activity; SB: Sedentary behaviors. Sedentary behaviors included watching television/video, playing computer.

Continued

| Cereal/wheat products | <2/d |
| --- | --- |
|  | ≥2/d |
| Meat/fish/  soybeans/egg | <2/d |
|  | ≥2/d |
| Fruits | ≥2/d |
|  | <2/d |
| Vegetables | ≥2/d |
|  | <2/d |
| Milk or yogurt | 1/d |
|  | <1/d |
| Fried chips/ cakes/cookies | No |
|  | Yes |
| Soft drink | No |
|  | Yes |
| Physical activity | ≥1h/d |
|  | <1h/d |
| Sedentary behaviors | <2h/d |
|  | ≥2h/d |
| Physical activity/  Sedentary behaviors | PA≥1h/d or SB<2h/d |
|  | PA<1h/d and SB≥2h/d |

　　　　　　　　　　　　　　　　　　　　　　　　　　　　　Overweight/obesity (OR) Metabolic syndrome　(OR)

S1 Fig. Association of rs12970134 with waist circumstance, body fat percentage, overweight/obesity and metabolic syndrome stratified by lifestyles

PA: Physical activity; SB: Sedentary behaviors. Sedentary behaviors included watching television/video, playing computer.
